# LNC_003296 regulates the proliferation and differentiation of ovine myoblasts by modulating the stability of Hsp70

**DOI:** 10.1080/01652176.2026.2720646

**Published:** 2026-08-25

**Authors:** Yize Song, Xiaofei Guo, Xiaosheng Zhang, Jinlong Zhang, Hui Sheng, Mingxing Chu

**Affiliations:** a State Key Laboratory of Animal Biotech Breeding, Institute of Animal Science, Chinese Academy of Agricultural Sciences, Beijing, China; b Tianjin Key Laboratory of Animal Molecular Breeding and Biotechnology, Tianjin Engineering Research Center of Animal Healthy Farming, Institute of Animal Science and Veterinary, Tianjin Academy of Agricultural Sciences, Tianjin, China

**Keywords:** Sheep, lncRNA, Hsp70, myoblasts, protein stability, LNC_003296, differentiation, skeletal muscle development

## Abstract

Long non-coding RNAs (lncRNAs) are involved in various regulatory processes during muscle development. Our previous RNA-seq study identified LNC_003296 as being significantly differentially expressed between ovariectomized Small-tailed Han sheep (OR-STH) and their non-ovariectomized counterparts (STH). Through biotinylated RNA pull-down assays and RNA immunoprecipitation (RIP), we further demonstrated that LNC_003296 directly interacts with Hsp70. Protein stability and degradation experiments revealed that LNC_003296 enhances the protein stability of Hsp70. Overexpression of either LNC_003296 or Hsp70 promoted myoblast proliferation, facilitated differentiation, and contributed to skeletal muscle fiber formation. Conversely, their knockdown suppressed cell proliferation and inhibited differentiation. In summary, these data indicate that LNC_003296 can bind to Hsp70 and enhance its protein stability, thereby influencing the proliferation and differentiation of myoblasts.

## Introduction

Sheep are highly valuable agricultural animals, and mutton is a staple meat in the global diet. To improve the meat production of existing sheep breeds, research focuses on the molecular mechanisms of skeletal muscle development in sheep. To identify genes affecting the muscle growth rate in sheep, several studies on ovine skeletal muscle growth have employed transcriptome sequencing (Zhang et al. 2014; Sun et al. 2016; Wei 2018; Tan 2025). To date, numerous lncRNAs have been identified in sheep muscle tissue (Chao 2016; Yuan 2020), where they play key roles in regulating muscle growth, development, and differentiation (Wei 2018). The discovery of different lncRNAs in ovine skeletal muscle will contribute to a better understanding of the regulatory functions of lncRNAs in sheep muscle growth.

Historically, long non-coding RNAs (lncRNA) genes were considered ‘junk DNA’. However, with advances in sequencing technologies, lncRNAs have been found to participate in many critical regulatory processes (Nagano and Fraser 2009; Klattenhoff 2013; Kopp and Mendell 2018). LncRNAs are defined as RNA transcripts longer than 200 nucleotides that lack protein-coding potential (Ponting et al. 2009; Shi et al. 2013). Recent studies indicate that lncRNAs play significant regulatory roles in the growth and development of skeletal muscle. lncRNA GTL2 is highly expressed during the differentiation of skeletal muscle satellite cells (SCs) and has been shown to regulate myogenesis in ewes by affecting the phosphorylation levels of PKA and CREB (Chen 2024). LNC-SEMT acts as a molecular sponge by antagonizing miR-125b to regulate IGF2 protein abundance both in vitro and in vivo (Wei 2018).

The lncRNAs exert their functions through various mechanisms, such as collaborating with RNA-binding proteins (RBPs) to form ribonucleoprotein complexes (RNPs). These RNPs are crucial for regulating processes such as RNA translation, nuclear export, stability, and splicing (Mo 2025). Researchers have found that lncRNAs can also exert their regulatory functions by directly binding to specific proteins and modulating their stability (Ma 2016; Sun 2021). A specific example is lncRNA-FKBP1C, which directly interacts with MYH1B to enhance its protein stability, thereby affecting myoblast proliferation and differentiation, as well as regulating skeletal muscle fiber-type transition (Yu et al. 2021).

Heat shock protein 70 (Hsp70) is a member of the highly conserved HSP molecular chaperone family, which plays a vital role in counteracting cellular stress and maintaining protein homeostasis (Clerico et al. 2015; Thakur et al. 2018). In skeletal muscle, Hsp70 is diffusely expressed in myocytes and nuclei, with its protein level doubling during the early stages of differentiation (Thakur 2019). This upregulation is functionally significant, as Hsp70 is crucial for regulating skeletal muscle homeostasis and regeneration. Specifically, the inhibition or downregulation of Hsp70 impairs the process of myoblast differentiation, underscoring its essential role in this critical cellular event (Fan et al. 2018).

In our previous study, we observed a significant difference in the expression of LNC_003296 in the longissimus dorsi muscle between ovariectomized and non-ovariectomized Small-tailed Han sheep (Chi 2025). This result suggests that LNC_003296 may influence muscle growth in sheep. The current study investigates the regulatory role of LNC_003296 in ovine skeletal muscle development, revealing that LNC_003296 binds to Hsp70 and functions by modulating its stability. Furthermore, the regulatory effects of the LNC_003296/Hsp70 interaction on myoblast proliferation and differentiation were examined, with downstream implications for Smad2 signaling.

## Materials and methods

### Animal samples collection

The experimental animals were sourced from the farm of Ulat Zhongqi, Bayannur City, Inner Mongolia Autonomous Region, China. The sheep flock was randomly divided into two groups: an ovariectomized group (*n* = 5, OR-STH) and a sham-operated group (*n* = 5, STH). No significant differences were observed between the two groups in terms of height, body weight, or age. Post-surgery, all sheep were housed in the same pen under uniform management conditions. They were maintained under a controlled 12-hour light/dark cycle with ad libitum access to water and a balanced diet provided under standardized conditions.

At the conclusion of the 6-month experimental period, the animals were slaughtered, and carcass weights were recorded (72.4 ± 1.86 kg and 88.4 ± 3.97 kg for the OR-STH and STH groups, respectively (*P* < 0.05). Samples of the *longissimus dorsi* muscle were collected, immediately flash-frozen in liquid nitrogen, and stored for subsequent total RNA extraction.

### Isolation and culture of ovine myoblasts

Longissimus dorsi muscle tissue was obtained from a 3-month-old STH sheep fetus. Following sequential rinses with 75% ethanol and PBS. For enzymatic digestion, the muscle tissue was minced into small pieces and digested with 0.25% trypsin (Solarbio, Beijing, China) at 4°C for 15 hours. Subsequently, the digestion mixture was transferred to an incubator and maintained at 37°C with 5% CO₂ for approximately 1 hour. The digested tissue was then filtered through 70 μm and 40 μm cell strainers. The isolated cells were seeded into 60 mm culture dishes and cultured in complete medium consisting of DMEM-F12 supplemented with 10% fetal bovine serum (FBS) and 1% penicillin/streptomycin. Once the cells reached over 90% confluence, they were transferred to 6-well plates for subsequent experiments. Myoblasts were then isolated via the differential adhesion method and identified through immunofluorescence staining.

Cells were maintained in a complete growth medium consisting of DMEM supplemented with 10% fetal bovine serum (FBS) and a 2% penicillin-streptomycin solution (100 μg/mL each). Cultures were incubated at 37°C in a humidified atmosphere containing 5% CO₂. To ensure experimental consistency, all assays were performed using cells prior to passage 4 (P4).

For immunofluorescence, cells were fixed with formaldehyde and incubated overnight at 4°C with primary antibodies (anti-MyoD1 and anti-Desmin (Abcam Technology, USA) 1:200 dilution). This was followed by a 2-hour room temperature incubation with secondary antibodies (Goat anti-Rabbit IgG (H+L) Highly Cross-Adsorbed Secondary Antibody, Thermo Scientific™, Waltham, MA, USA, 1:500 dilution). Nuclei were subsequently counterstained with DAPI (1:500 dilution) for 3 minutes at room temperature.

### Fluorescence in situ hybridization (FISH)

To detect the subcellular localization of LNC_003296 in ovine myoblasts, RNA fluorescence in situ hybridization (FISH) was performed using a commercial FISH kit (GenePharma, Shanghai, China) and a Cy3-labeled LNC_003296 probe mixture, following the manufacturer's instructions. The probe was used at a concentration of 2 μM in a total volume of 100 μL, denatured at 73°C for 5 minutes, and then added to the culture dish. Hybridization was carried out overnight in a 37°C incubator under dark conditions. Negative control (NC), 18S probes were used as references for nuclear and cytoplasmic compartments, respectively. Images were captured using an LSM 710 laser scanning confocal microscope. The probe sequences are detailed in Table S1.

### Plasmid construction and cell transfection

siRNAs targeting LNC_003296 and Hsp70 were designed based on the provided sequences (Table S2). Simultaneously, overexpression plasmids for these two genes were constructed by inserting LNC_003296 (Supplementary 1) and the CDS sequences of Hsp70 into the pcDNA3.1 vector, respectively. The overexpression vectors and siRNAs were synthesized by GenePharma (Shanghai, China).

Well-conditioned myoblasts were seeded into 6-well plates and transfected when cell confluence reached approximately 70%. For transfection, the complete medium in the wells was replaced with 1.5 mL/well of OPTI-MEM medium and incubated at 37°C for 30 minutes. Solution A was prepared by mixing 8 µL of the plasmid for transfection with 250 µL of OPTI-MEM. Solution B was prepared by mixing 8 µL of Lipofectamine 2000 (Invitrogen, USA) with 250 µL of OPTI-MEM. Solutions A and B were thoroughly mixed and allowed to stand at room temperature for 20 minutes before being added to the 6-well plates. After 5 hours, the medium was replaced with complete medium for subsequent experiments.

### Isolation of nuclear and cytoplasmic RNA

Ovine myoblasts were cultured in 10 cm^2^ dishes until they reached approximately 90% confluence. The cells were then gently washed twice with PBS buffer and subsequently harvested by scraping into centrifuge tubes.

Nuclear and cytoplasmic RNA were separated using the NE-PER Nuclear and Cytoplasmic Extraction Reagents (Thermo Scientific™, Waltham, MA, USA), strictly following the manufacturer's protocol. After quantification, the RNA samples were stored at −80°C for subsequent experiments.

### EdU assay and cell counting kit-8 (CCK-8) assay

Ovine myoblasts were seeded into 6-well plates and cultured overnight. When cell confluence reached approximately 70%, a 10 µM EdU working solution (Beyotime, Ltd., Shanghai, China) was added to each well, followed by incubation at 37°C for 2 hours. After incubation, the medium was removed, and the cells were washed 1–2 times with PBS. The cells were then fixed with 4% paraformaldehyde for 30 minutes and permeabilized with PBS containing 0.5% Triton X-100 for 10 minutes. Finally, a reaction solution was added to each well, and the cells were incubated at room temperature in the dark for 30 minutes. Nuclei were counterstained with DAPI, and the number of EdU-positive cells was assessed using a fluorescence microscope.

Myoblasts were seeded into a 96-well plate and cultured overnight at 37°C. When cell confluence reached approximately 70%, 10 µL of CCK-8 reagent (Solarbio, Beijing, China) was added to each well according to the manufacturer's instructions. Absorbance at 450 nm was measured at 0, 6, 12, 24, 36, and 48 hours to assess cell proliferation.

### Quantitative real-time PCR (RT-qPCR)

Total RNA (1 μg per sample) was first reverse transcribed into cDNA using a commercial RT reagent (TaKaRa, Japan). Quantitative PCR was then performed on a LightCycler 480II system (Roche, Basel, Switzerland) using SYBR Premix Ex Taq II for fluorescence detection, with GAPDH as the endogenous reference gene for normalization (Table S2). The relative expression of target genes was calculated using the 2^−ΔΔCt^ method. All primers were designed with Premier 5 software and synthesized by Sangon Biotech (Beijing, China).

### Western blot

Proteins were extracted from myoblasts using a protein extraction kit (Beyotime, Ltd., Shanghai, China), following the manufacturer's protocol. Protein concentration was subsequently determined with the Bicinchoninic Acid (BCA) Protein Assay Kit (Beyotime, Ltd., Shanghai, China) using a standard procedure.

The extracted proteins were separated by molecular weight via SDS-Polyacrylamide Gel Electrophoresis (SDS-PAGE) and then transferred onto PVDF membranes. Prior to antibody incubation, the membranes were blocked at room temperature for 1 hour with blocking buffer (Beyotime, Ltd., Shanghai, China). They were then incubated overnight at 4°C with primary antibodies diluted in dilution buffer (Beyotime, Ltd., Shanghai, China). The primary antibodies and their dilutions were as follows: anti-CCND1 (Cell Signaling Technology, USA) 1:3000, anti-CDK4 (Cell Signaling Technology, USA) 1:3000, anti-Hsp70 (Abcam Technology, USA) 1:4000, anti-MyHC-I (Abcam Technology, USA) 1:5000, anti-MyoD (Abcam Technology, USA) 1:1000, and anti-GAPDH (Abcam Technology, USA) 1:20000.

Following primary antibody incubation, the membranes were incubated with corresponding secondary antibodies (Goat anti-Rabbit IgG, Abcam Technology, USA) for 2 hours at room temperature. Secondary antibodies were diluted using secondary antibody dilution buffer (Beyotime, Ltd., Shanghai, China). Finally, protein bands were visualized and analyzed using the Odyssey CLX imaging system (Li-COR, USA) with a chemiluminescent substrate. Each experimental group included at least three independent replicates.

### Pull-down of biotinylated RNA Probes, GO and KEGG enrichment analysis

Biotin-labeled RNA probes (Table S3) were synthesized by GenePharma (Shanghai, China) and bound to magnetic beads. RNA-protein complexes were isolated using the PierceTM Magnetic RNA-Protein Pull-Down Kit (Thermo Scientific™, Waltham, MA, USA). The recruited proteins were subsequently analyzed either by mass spectrometry (LC-MS) or western blotting.

For proteins identified by LC-MS, functional annotation was performed through Gene Ontology (GO) and Kyoto Encyclopedia of Genes and Genomes (KEGG) enrichment analyzes. GO terms and KEGG pathways with a Q-Value of ≤0.05 were defined as significantly enriched.

### Silver staining

Following the pull-down assay, the obtained proteins were separated by SDS-PAGE and subsequently stained using the Fast Silver Staining Kit (Beyotime, Ltd., Shanghai, China).

### RNA immunoprecipitation (RIP)

Ovine myoblasts were collected and lysed using the provided lysis buffer. RNA-protein complexes were immunoprecipitated according to the manufacturer's protocol of the BeyoRIPTM RIP Assay Kit (Beyotime, Ltd., Shanghai, China), using specific antibodies including an Hsp70 antibody (Abcam Technology, USA) and an ovine IgG antibody (Beyotime, Ltd., Shanghai, China). The co-precipitated RNA was subsequently recovered using the RNAeasy^TM^ Animal RNA Isolation Kit (Beyotime, Ltd., Shanghai, China) and analyzed by RT-PCR. In the immunoprecipitation experiments, IgG served as the negative control for LNC_003296, while PCNA was used as the negative control for Hsp70.

### Data analysis

All statistical analyzes were conducted using GraphPad Prism 9.5.1 (GraphPad Software, San Diego, CA, USA). Differences between two groups were assessed using an unpaired Student's *t*-test. For comparisons involving more than two groups, one-way analysis of variance (ANOVA) was performed, followed by Tukey's honestly significant difference (HSD) test for post hoc pairwise comparisons. Data are presented as the mean ± standard error of the mean (SEM), with statistical significance indicated as *P < 0.05, **P < 0.01 and ***P < 0.001. A minimum of three biological replicates were included for all experiments, with each biological replicate subjected to three technical replicates.

## Results

### Biological characteristics of LNC_003296

**Figure 1.**
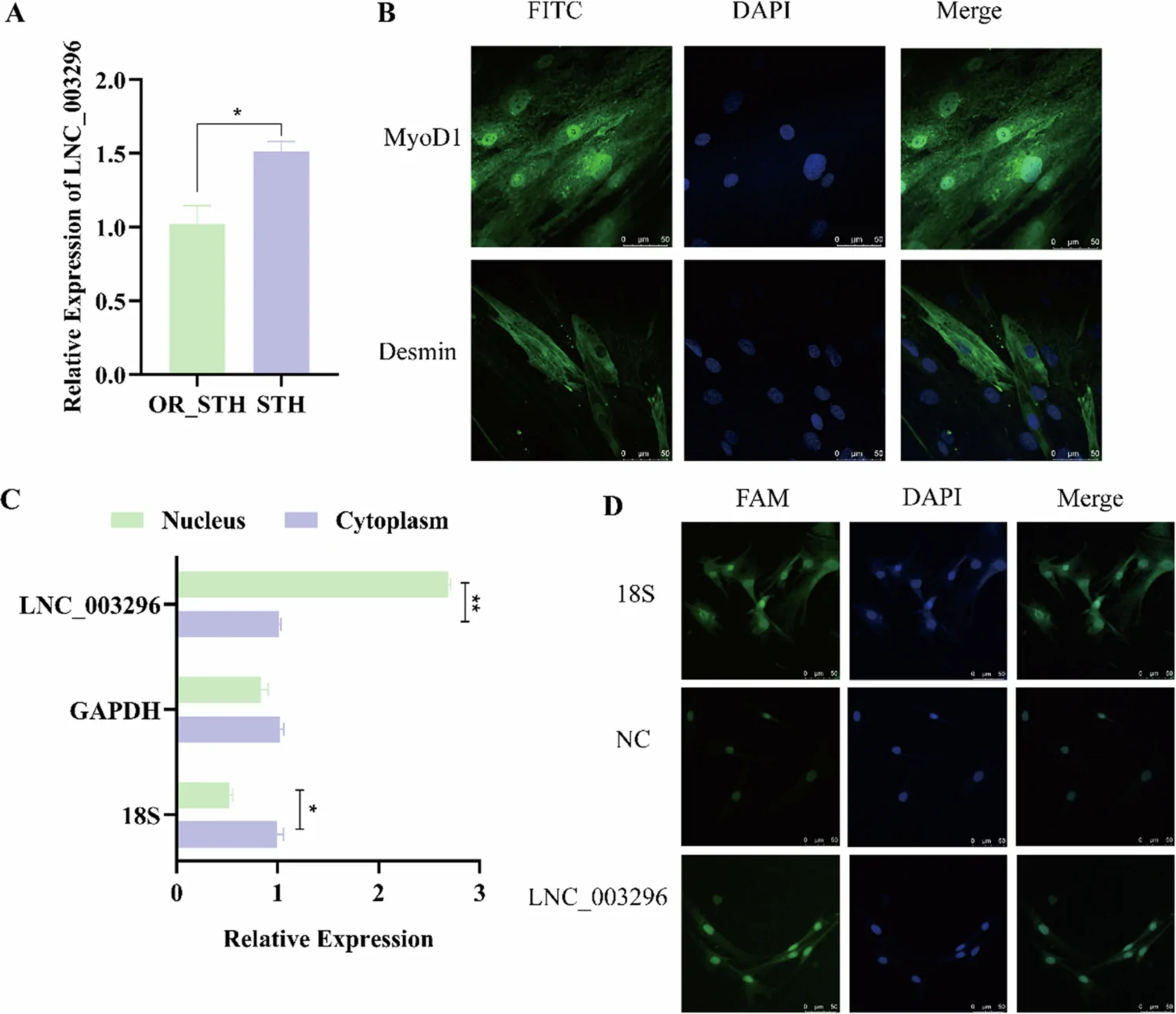
Biological characteristics of LNC_003296. (A) Expression level of LNC_003296 in the longissimus dorsi muscle of ovariectomized and sham-operated sheep; (B) Immunofluorescence identification of sheep myoblasts; (C) Subcellular expression level of LNC_003296; (D) Subcellular localization of LNC_003296 detected by immunofluorescence (Mean values ± SEMs, *n* = 3, *P < 0.05, **P < 0.01, ns, not significant).

Based on our previous RNA-seq data of *Longissimus dorsi* muscles from ovariectomized and non-ovariectomized Small-tailed Han sheep, we identified lncRNA LNC_003296 as significantly upregulated in the non-ovariectomized group. Subsequent RT-qPCR validation confirmed that its expression pattern across different experimental groups was fully consistent with the sequencing results (Figure 1A). Meanwhile, myoblasts were isolated and cultured from *Longissimus dorsi* muscle of Small-tailed Han sheep. Immunofluorescence staining confirmed the specific expression of myogenic marker proteins MyoD1 and Desmin in the isolated cells, demonstrating their identity as myoblasts (Figure 1B).

We predicted the subcellular distribution of LNC_003296 using an online lncRNA localization prediction tool, which indicated predominant nuclear localization. Subsequent RNA nucleo-cytoplasmic fractionation assays revealed significantly higher abundance of LNC_003296 in the nuclear fraction compared to the cytoplasmic fraction (Figure 1C). This finding was further confirmed by RNA fluorescence in situ hybridization (FISH) analysis (Figure 1D).

### LNC_003296 promotes myofibroblast proliferation and differentiation

**Figure 2.**
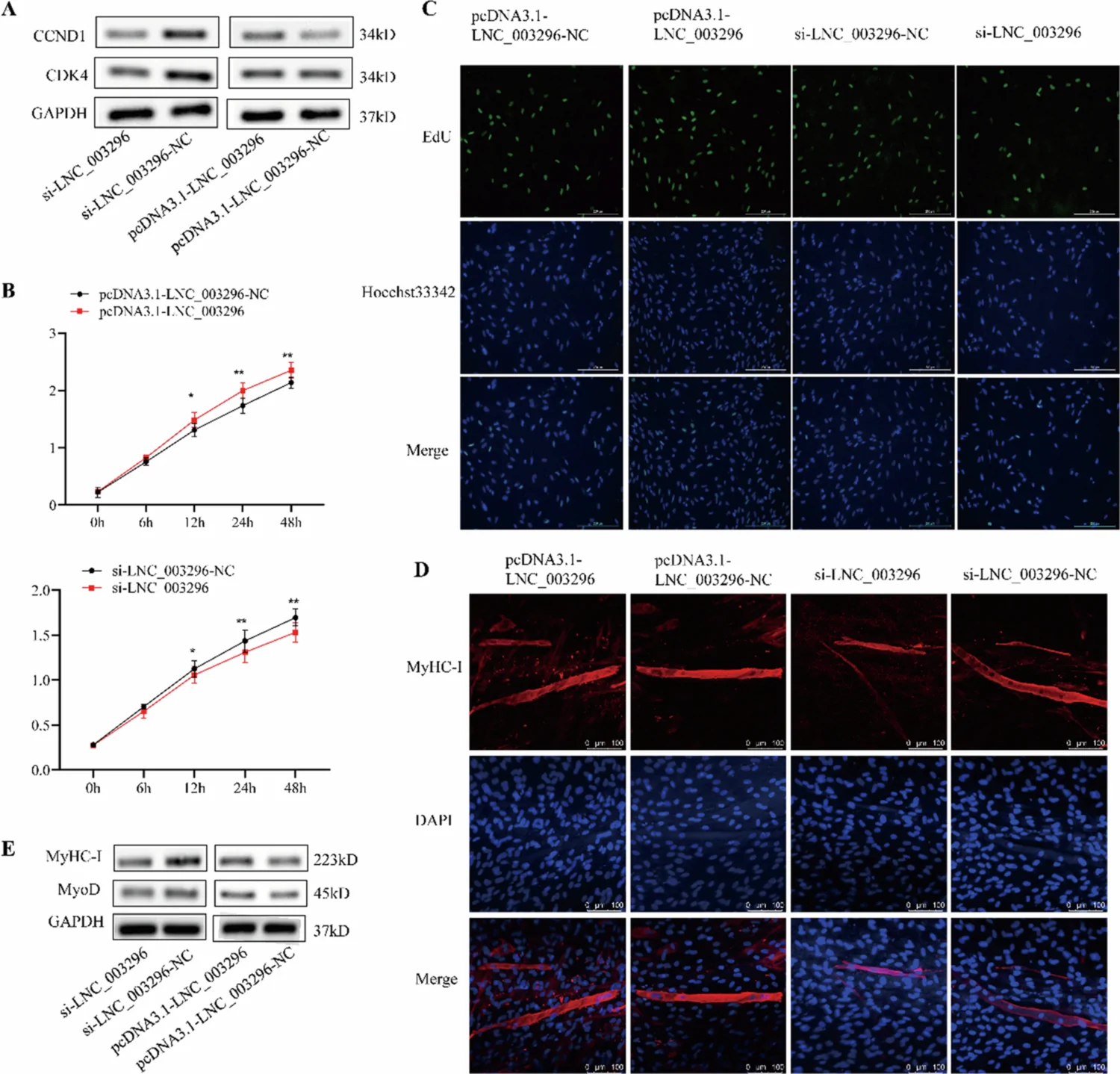
LNC_003296 promotes the proliferation and differentiation of myoblasts. (A) Detection of proliferation factor expression levels by WB; (B) Detection of cell viability by CCK-8 assay; (C) Detection of cell proliferation efficiency by EDU assay; (D) LNC_003296 promotes myotube formation; (E) Detection of differentiation factor expression levels by WB.

To explore the biological role of LNC_003296, we transfected myoblasts with plasmids for its overexpression or knockdown and evaluated the mRNA and protein levels of key proliferation markers using RT-qPCR and western blot. As shown in Figure S1A, overexpression of LNC_003296 led to a marked increase in *CDK4* and *CCND1* expression compared with the control, whereas their levels decreased upon LNC_003296 knockdown. Consistent with the mRNA results, the protein expression of CDK4 and CCND1 followed the same pattern (Figure 2A, S1B).

We further assessed the effect of LNC_003296 on myoblast proliferation using CCK-8 and EdU assays. The CCK-8 results indicated that cell viability was significantly elevated at 12, 24, and 48 hours after LNC_003296 overexpression relative to the pcDNA3.1-NC group (Figure 2B). Furthermore, EdU staining revealed a lower percentage of EdU-positive cells upon LNC_003296 knockdown (Figure 2C), consistent with the conclusion that LNC_003296 promotes cell proliferation.

We also investigated whether LNC_003296 influences myoblast differentiation. Immunofluorescence analysis demonstrated a clear increase in myotube formation upon LNC_003296 overexpression, while myotube development was impaired after its knockdown (Figure 2D). Accordingly, RT-qPCR showed that the differentiation markers *MyHC-I* and *MyoD* were upregulated when LNC_003296 was overexpressed, but downregulated when it was knocked down (Figure S1C). Western blot data further confirmed this expression trend (Figure 2E, Figure S1D).

### LNC_003296 is able to bind to protein Hsp70

**Figure 3.**
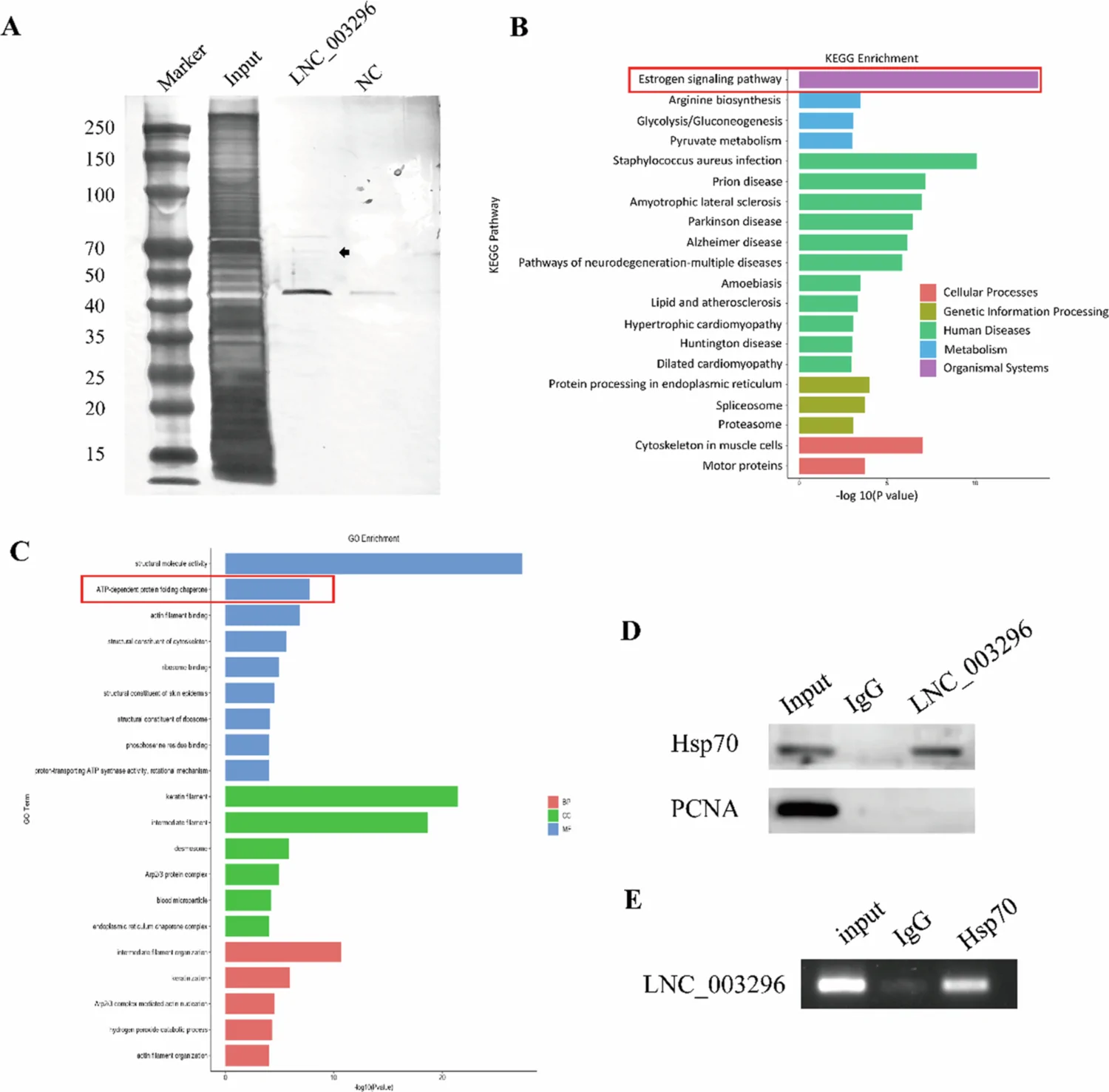
The binding protein of LNC_003296 is Hsp70. (A) Results of RNA and mass spectrometry analysis after in vitro protein precipitation followed by SDS-PAGE and silver staining; (B, C) KEGG and GO pathway analysis of proteins identified by mass spectrometry; (D) Validation of the specific interaction between LNC_003296 and Hsp proteins by Western blot (WB); (E) Reverse validation using RIP technique with biotin-labeled RNA probe precipitation results (Mean values ± SEMs, *n* = 3, *P < 0.05, **P < 0.01, ns, not significant).

To comprehensively investigate the biological regulatory mechanisms of LNC_003296 in sheep myoblasts, we performed natural RNA pull-down assays coupled with mass spectrometry analysis to identify LNC_003296-interacting proteins (Supplementary 2). The RNA pull-down products were separated by SDS-polyacrylamide gel electrophoresis (SDS-PAGE), with subsequent silver staining revealing distinct protein bands, demonstrating the specific protein binding capacity of LNC_003296 (Figure 3A). The putative interacting proteins identified by liquid chromatography-mass spectrometry (LC-MS/MS) were subsequently subjected to functional classification using the Kyoto Encyclopedia of Genes and Genomes (KEGG) pathway database and Gene Ontology (GO) enrichment analysis. Notably, the KEGG enrichment analysis demonstrated significant enrichment in the estrogen signaling pathway (*P* < 0.001) (Figure 3B, Figure S3A). Concurrently, GO enrichment analysis revealed the inclusion of ‘ATP-dependent protein folding chaperone’ (GO:0140662) as a significantly enriched term in the Molecular Function category (Figure 3C, Figure S3B). Based on these convergent findings, we have identified heat shock protein 70 (Hsp70) as a key molecular chaperone and functional protein of interest.

To confirm the physical interaction between LNC_003296 and Hsp70, a Western blot test was performed using an antibody against Hsp70 protein. The results demonstrated specific enrichment of Hsp70 in the LNC_003296 probe pull-down samples compared to the IgG control group (Figure 3D). To reciprocally validate the RNA pull-down results, we performed RIP assays. The results showed that LNC_003296 was significantly more enriched in samples immunoprecipitated with anti-Hsp70 antibody compared to those immunoprecipitated with sheep IgG (Figure 3E). These results demonstrate a physical interaction between LNC_003296 and Hsp70, suggesting that LNC_003296 may regulate myoblast proliferation and differentiation through its modulation of Hsp70 function.

### LNC_003296 inhibits Hsp70 degradation

**Figure 4.**
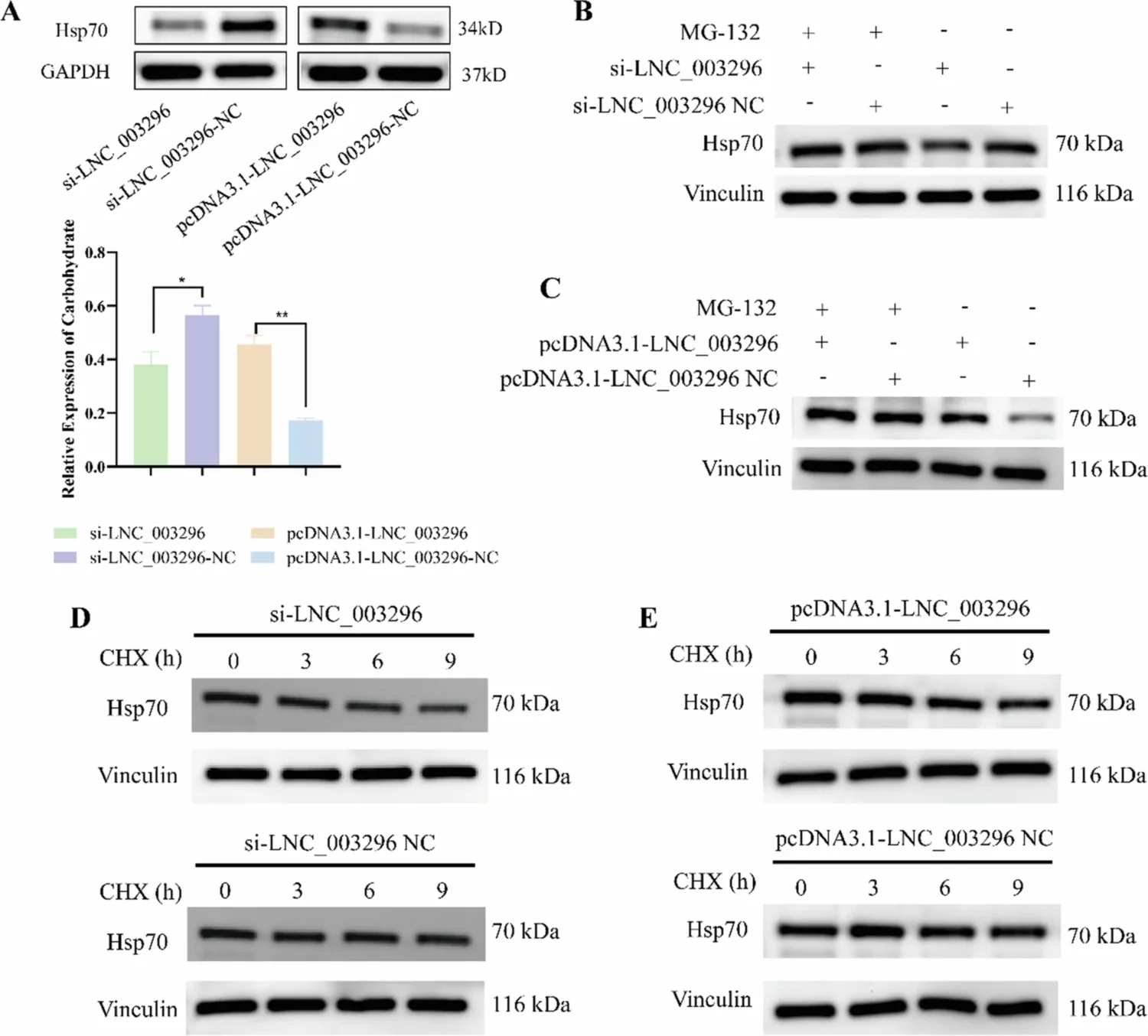
LNC_003296 inhibits the degradation of Hsp70. (A) The effect of LNC_003296 on the expression level of Hsp70 protein; (B, C) MG-132 rescues the Hsp70 level decline caused by LNC_003296 knockdown; (D, E) LNC_003296 significantly affects the stability of Hsp70 in myoblasts (Mean values ± SEMs, *n* = 3, *P < 0.05, **P < 0.01, ns, not significant).

Based on the binding between LNC_003296 and Hsp70, we evaluated the functional impact of their interaction on Hsp70. The results showed that knocking down LNC_003296 in sheep myoblasts significantly reduced Hsp70 expression (Figure 4A). Concurrently, overexpression of LNC_003296 rescued the decline in Hsp70 levels. These data indicate that LNC_003296 may modulate Hsp70 protein levels through a specific mechanism. CHIP functions as an E3 ubiquitin ligase that ubiquitinates Hsp70/HSC70 via its U-box domain. The increased stability of Hsp70 upon CHIP knockdown in mice further demonstrates its role in regulating protein degradation in vivo. Therefore, we hypothesized that the observed effects might be attributable to proteasomal degradation and further investigated the role of LNC_003296 in the proteasomal degradation of Hsp70. In sheep myoblasts, the proteasomal inhibitor MG-132 rescued the decrease in Hsp70 resulting from LNC_003296 knockdown (Figure 4B). On the other hand, LNC_003296 overexpression produced a synergistic effect with MG-132, leading to a further accumulation of Hsp70 (Figure 4C). Next, we added Cycloheximide to the culture medium to explore the inhibitory effect of LNC_003296 on Hsp70 protein degradation. The results showed that LNC_003296 knockdown significantly affected Hsp70 stability in myoblasts by shortening its half-life (Figure 4D), while LNC_003296 overexpression produced the opposite effect (Figure 4E).

### Hsp70 promotes myoblast proliferation and differentiation

**Figure 5.**
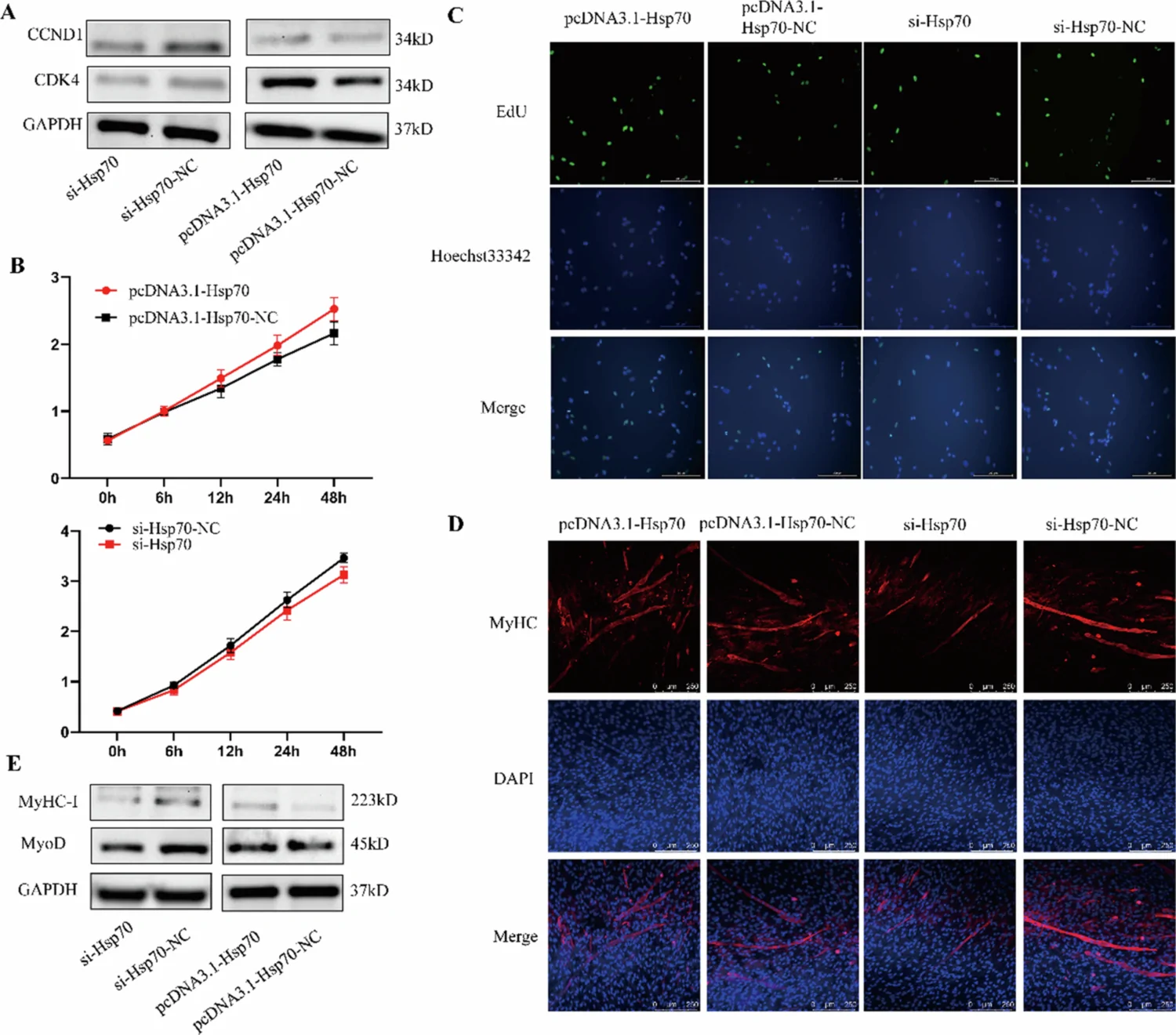
Hsp70 promotes myoblast proliferation and differentiation. (A) Detection of proliferation factor expression levels by WB; (B) Detection of cell viability by CCK-8 assay; (C) Detection of cell proliferation efficiency by EdU assay; (D) Hsp70 promotes myotube formation; (E) Detection of differentiation factor expression levels by WB.

To investigate the biological function of Hsp70, gain- and loss-of-function studies were performed in myoblasts via plasmid-mediated overexpression and knockdown. Expression changes in proliferation-related markers were analyzed at both transcriptional and translational levels by RT-qPCR and western blot. As depicted in Figure S3A, Hsp70 knockdown resulted in a significant downregulation of *CDK4* and *CCND1* mRNA, which was conversely enhanced by Hsp70 overexpression. A parallel trend was observed at the protein level, confirming the regulatory influence of Hsp70 on these key cell cycle regulators (Figure 5A, Figure S3B).

The pro-proliferative role of Hsp70 was further substantiated by functional assays. CCK-8 analysis revealed a marked increase in cell viability following Hsp70 overexpression at 12, 24, and 48 hours post-transfection, compared to the control group (Figure 5B). In line with this, EdU incorporation assays displayed a notable reduction in the proportion of EdU-positive cells upon Hsp70 silencing (Figure 5C), collectively indicating that Hsp70 facilitates myoblast proliferation.

Furthermore, the effect of Hsp70 on myogenic differentiation was examined. Immunofluorescence staining revealed that myotube formation was potently induced by Hsp70 overexpression, whereas Hsp70 deficiency markedly suppressed this process (Figure 5D). Consistent with the morphological observations, the mRNA expression of the differentiation markers MyHC-I and MyoD was elevated in Hsp70-overexpressing cells but diminished following its knockdown (Figure S3 C). This regulatory pattern was further validated at the protein level by western blot analysis (Figure 5E, Figure S3D).

### Hsp70 prevents receptor-dependent Smad2 phosphorylation

**Figure 6.**
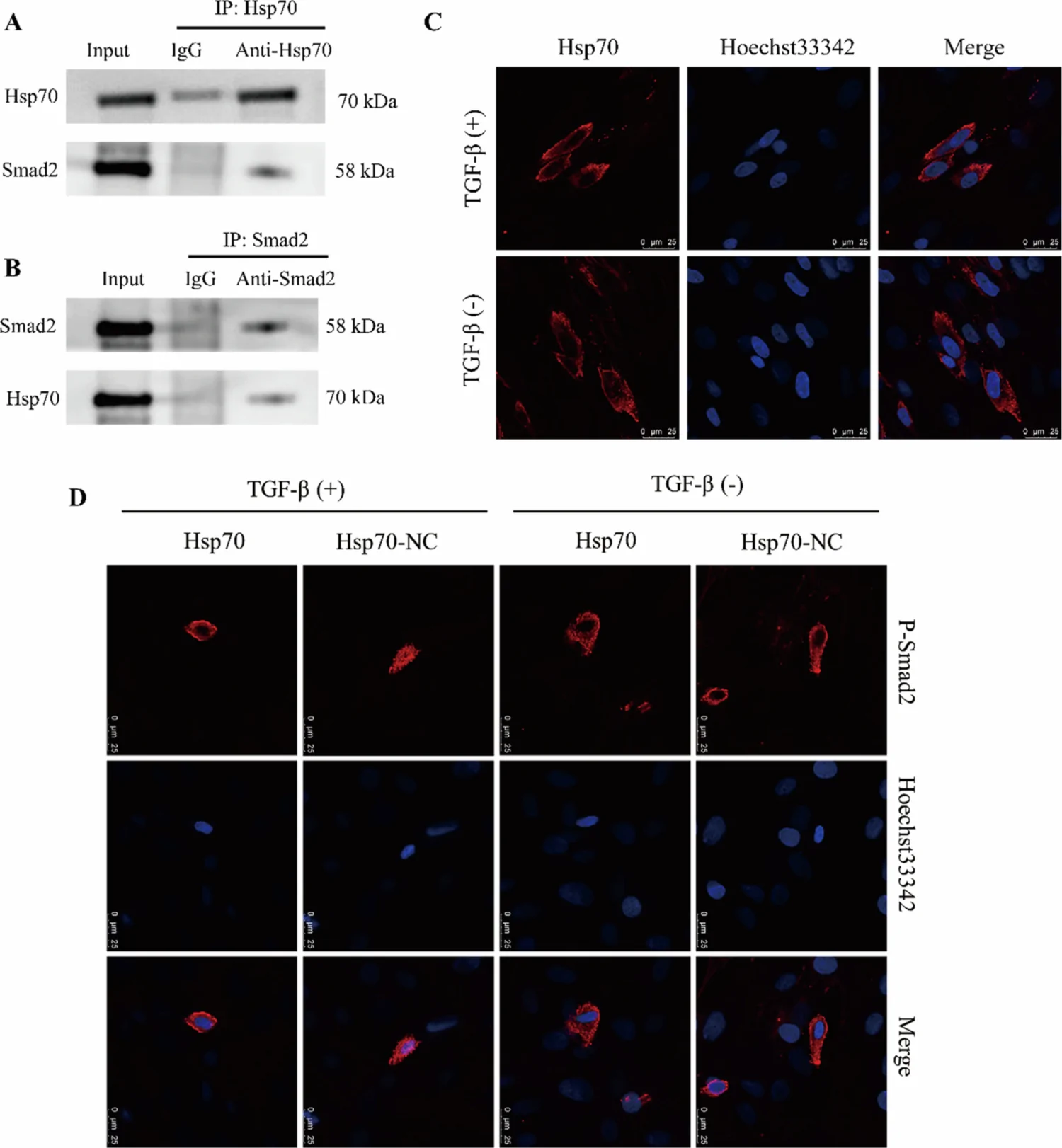
Hsp70 inhibits receptor-dependent Smad2 phosphorylation. (A, B) Specific binding of Hsp70 with Smad2; (C) TGF-*β* has no effect on the subcellular localization of Hsp70; (D) Hsp70 hinders TGF-*β*-induced nuclear translocation of Smad2.

Hsp70, as a molecular chaperone, can recognize and bind to a variety of proteins. Multiple studies have shown that Hsp70 can bind to Smad2 and influence its biochemical functions. To confirm the interaction between Hsp70 and Smad2, we performed co-immunoprecipitation assays using their respective antibodies. The results showed that when Hsp70 was used for immunoprecipitation, Smad2 was detected in the precipitates by Western Blot (Figure 6A). Conversely, when Smad2 was used for immunoprecipitation, Hsp70 was also detected (Figure 6B). This interaction was specific, as no signals for Hsp70 or Smad2 were observed in the negative control samples immunoprecipitated with IgG. These results clearly demonstrate that Hsp70 and Smad2 can form a complex in sheep myoblasts. The interaction between Hsp70 and Smad2 suggests that Hsp70 may also play a role in TGF-*β* signal transduction. Smad2 is an intracellular mediator of TGF-*β* signaling. It is directly phosphorylated by the activated type I receptor kinase, then shuttles from the cytoplasm into the nucleus to regulate the expression of target genes. To investigate whether the inhibitory function of Hsp70 on TGF-*β* signaling is mediated through its interaction with Smad2, we first examined the subcellular localization of Hsp70 with or without TGF-*β* stimulation. In the absence of TGF-*β* stimulation, Hsp70 was localized in the cytoplasm of sheep myoblasts, and this localization remained unchanged following TGF-*β* treatment (Figure 6C). Upon phosphorylation, Smad2 translocates from the cytoplasm to the nucleus. However, we found that TGF-*β*-induced nuclear translocation of Smad2 was significantly impeded by Hsp70 overexpression (Figure 6D). These results suggest that Hsp70 exerts its inhibitory activity in the cytoplasm, likely by preventing the phosphorylation of Smad2. Taken together, our Co-IP (Figure 6A and B) and immunofluorescence (Figure 6C and D) data demonstrate that Hsp70 interacts with Smad2 in the cytoplasm and inhibits its TGF-*β*-induced nuclear translocation, suggesting that Hsp70 likely impedes Smad2 phosphorylation.

## Discussion

An increasing number of lncRNAs have been identified as critical regulators of myoblast proliferation and differentiation (Chao 2018; Cao 2025; Liu et al. 2026; Yang 2026). For instance, lncRNA-SSYSL has been characterized as a suppressor of muscle development, playing a key role in PRC2-mediated myogenesis (Jin 2018). LncRNA-Six1 exerts cis-regulation on its cognate protein-coding gene *Six1*, thereby promoting cell proliferation and contributing to muscle growth (Cai 2017). In this study, we initially characterized the function of LNC_003296 in the proliferation and differentiation of ovine myoblasts. Specifically, overexpression of LNC_003296 significantly increased the EdU-positive rate and enhanced cell viability. Mechanistically, LNC_003296 promoted myoblast proliferation by upregulating the mRNA and protein expression levels of CCND1 and CDK4. Concurrently, it facilitated myoblast differentiation and fusion by elevating the expression of MyHC-Ⅰ and MyoD. Collectively, these data demonstrate that LNC_003296 overexpression promotes both the proliferation and differentiation of ovine myoblasts into myofibers.

LncRNAs exert their biological functions through diverse mechanisms (McFadden and Hargrove 2016; Muniz 2022). It has been found that they can operate by binding to proteins and modulating their stability (Ferrè et al. 2016; Wang et al. 2025). For example, lncRNA AGPG binds to PFKFB3, enhances its stability, and subsequently activates glycolytic flux to promote cell cycle progression (Liu 2020). In broiler chickens, lncRNA-FKBP1C interacts with the protein MYH1B to stabilize it; its overexpression inhibits myoblast proliferation, promotes differentiation, and participates in skeletal muscle fiber formation (Yu et al. 2021). In the present study, we further demonstrated through pull-down assays and RIP that LNC_003296 directly interacts with Hsp70. Protein stability and degradation experiments revealed that LNC_003296 enhances the protein stability of Hsp70. These results suggest that in ovine myoblasts, LNC_003296 likely regulates myoblast function by modulating the protein stability of Hsp70.

Studies have shown that heat shock proteins (HSPs) play a crucial role in mitigating cellular stress and maintaining protein homeostasis. Notably, Hsp70 overexpression has been demonstrated to enhance muscle regeneration and functional recovery in both atrophy and freeze-injury models (McArdle et al. 2004; Miyabara et al. 2006). In C2C12 cells, plasmid-mediated Hsp70 overexpression does not alter the proliferation or differentiation rate; however, it increases myotube width in differentiated cells, indicating that elevated Hsp70 expression promotes myoblast fusion (Gehrig 2012; Baumann and Otis 2015; Baumann et al. 2016; Thakur 2020). Importantly, the aforementioned studies on Hsp70 were conducted using mature murine muscle, leaving its regulatory role in ovine myoblasts unclear. Our research specifically investigated the impact of Hsp70 on the proliferation and differentiation of sheep myoblasts. The results revealed that knockdown of Hsp70 in ovine myoblasts led to decreased cell viability and a significant reduction in myotube formation, while its overexpression produced the opposite effects. This aligns with the established function of muscle precursor cells, which can fuse to form new myofibers or integrate into damaged ones to facilitate repair and maintain muscle homeostasis.

In this study, we characterized the interaction between Hsp70 and SMAD2, both recognized as key regulators in skeletal muscle proliferation and differentiation. Specifically, Hsp70 protein is diffusely expressed in both the cytoplasm and nucleus of C2C12 myoblasts, and its levels double during the early differentiation phase (Thakur 2019). Similarly, we examined the subcellular localization of the Hsp70 protein in ovine myoblasts. The results indicate that Hsp70 is expressed in the cytoplasm, and its expression is not affected by TGF-*β* treatment. The TGF-*β* family constitutes a group of secreted polypeptides that regulate a variety of developmental and biological processes, including cell proliferation, differentiation, apoptosis, and development (Li et al. 2011). The activation and termination of Smad2 activity are critical steps in TGF-*β* signal transduction. Smad2 is predominantly cytoplasmic; upon ligand stimulation, *p*-Smad2 and Smad4 accumulate in the nucleus. Hsp70 interacts with Smad2 in the cytoplasm, preventing its receptor-dependent phosphorylation and nuclear translocation in response to TGF-*β* stimulation (Chen et al. 2006; Li et al. 2011). Functionally, TGF-*β* inhibits the myogenic program by activating Smad2 and Smad3, which directly bind to myogenic transcription factors such as MyoD and Myogenin, recruiting transcriptional repressor complexes to functionally suppress their activity (Liu et al. 2001; Rebbapragada et al. 2003). The aforementioned studies demonstrate that high expression of Hsp70 can effectively block TGF-*β*-induced phosphorylation of Smad proteins, thereby conferring resistance to TGF-*β* stimulation.

Although our data demonstrate that LNC_003296 physically interacts with Hsp70 and regulates its stability, and both genes share similar functional effects on myoblast proliferation and differentiation, we acknowledge that formal rescue experiments (e.g. Hsp70 overexpression in LNC_003296 knockdown cells) would provide more direct genetic evidence for their epistatic relationship. Furthermore, while we show that Hsp70 interacts with Smad2 and inhibits its phosphorylation and nuclear translocation (Figure 6), the direct effect of LNC_003296 on Smad2 phosphorylation has not been experimentally tested in the current study. Therefore, the proposed connection between LNC_003296 and Smad2 remains inferential, based on the established LNC_003296–Hsp70 interaction and the known Hsp70–Smad2 regulatory relationship. Future studies addressing both the rescue experiments and the direct LNC_003296–Smad2 link will be necessary to fully validate the mechanistic hierarchy of this proposed regulatory cascade.

## Supplementary Material

Fig_S2.tif Fig_S2.tif

Fig_S3.tif Fig_S3.tif

Fig_S1.tif Fig_S1.tif

Supplementary Table.docx Supplementary Table.docx

Supplementary 1.docx Supplementary 1.docx

Supplementary Figure.docx Supplementary Figure.docx

Supplementary 2.xlsx Supplementary 2.xlsx

## Data Availability

The manuscript has no associated data.
