# Supplementary material for: LNC_003296 regulates the proliferation and differentiation of ovine myoblasts by modulating the stability of Hsp70
Source: Vet Q. 2026 Aug 25;46(1):2720646. doi: 10.1080/01652176.2026.2720646 (PMC13520862; doi:10.1080/01652176.2026.2720646)
Supplement: Supplementary file 1 [file TVEQ_A_2720646_SM6629.docx]

**Fig.1 LNC_003296 promotes the proliferation and differentiation of myoblasts. (A)** mRNA expression levels of cell proliferation factors; **(B)** Protein expression levels of cell proliferation factors; **(C)** mRNA expression levels of cell differentiation factors; **(D)** Protein expression levels of cell differentiation factors.

**Fig.2 KEGG and GO pathway analysis of proteins identified by mass spectrometry. (A)** KEGG; **(B)** GO.

**Fig.3 Hsp70 promotes the proliferation and differentiation of myoblasts. (A)** mRNA expression levels of cell proliferation factors; **(B)** Protein expression levels of cell proliferation factors; **(C)** mRNA expression levels of cell differentiation factors; **(D)** Protein expression levels of cell differentiation factors.
