# Supplementary material for: LNC_003296 regulates the proliferation and differentiation of ovine myoblasts by modulating the stability of Hsp70
Source: Vet Q. 2026 Aug 25;46(1):2720646. doi: 10.1080/01652176.2026.2720646 (PMC13520862; doi:10.1080/01652176.2026.2720646)
Supplement: Supplementary file 2 [file TVEQ_A_2720646_SM6630.docx]

>LNC_003296

ACGGTATGTAAGCAGCTGGATTTATATCTTCTGCTGCCACAGGGATGGGCTCTTCTGTTTACTTAATGCAGTCATGGAACAGGAGCACAAAATGCCATTCTACGATGTAGACTGGGGCTAGAAACCCAGAAAAGACACAGCCCCACAGTTATTAGTCTATGGGGGTGGACACAGAGGAAGTGGGAGGGAAAACAAAACCCTTCTCCTCAAAGTGAGCCTGCCAGCTGATATTTCAAAACATGTAAAGAAACCTAAGGCTAAGAAGACAACCCACCAaaacaacaaatgaaataaattcaGTTGAAATGAAACTGACTTCATGGGACAATCTGAAGGAGTGATATAAGTAACTATTAACTATAAGAAGGAGAACTATTTGGGATAAAACATGTAAAAACTAAACAGTCATAAAACTGCCAGGCAGTGTTTAATGCATGGTTATGGCATGCATAGGTTCTTGTGCTCAAAGTGAGGATAAAACTACTGGGAAATTTATAGTTAAGCAAATATGTTAAACATGCAGGATAATATTAATAAAGTATAGAAAAATAATGTCTAGTTTCTAAatcaacagaaggaaaaaagaggtttatagaaaattttattaatCCAACAGgacaggaaatgagaaaaaaagaattagaggGAAAAGGTGCTAAAAGGAGATTTCTACCCATGTAAATTATTGAATTGTGCTATTGAATTATCAGACAATTTTCCCCAGGATCTCTAGTCCACAAGTAGTGATACTTAACAACTGAGTGAGTAGGGGTAGGGGGTTGATTTGTATTGTTCATTGATTTTACTGATTTCCATGGTATAAGTACTCCTACCTTGACCAATTTCAAGATACCAGTGTAACATCAGTCAGTTCACAAAATCCTTGAAAATGTAACAGTAATATATAGGGAGCTTGCGCTTGCTGACTTTAGCATACTGCTGTTTGTTTGGGAAAGACAGGCTTTTCTCCATGGTCTTCATACTATATATAATTTTGACAACAGAATAAAGATTTAGAAAACCCAGATTCTCTGCATTACTTCTCTAACTGAAACCATGTAGAAATTTATTGGGAAACCAGGAGTAGGTGGCATTCTGTTATTAGAGTATTTATCATATGGTTATGGAAGGCTAGTAATTTATGATCCTTGCTAGAAAAGCCAGATCAATTCACAGATAAATGTCCTCAGCACAATAGAATTTATATCATCCATGTGATAATTGACCACTGTGTCCTGCATTCAATCATTCATCTCATCTGATAAAATGAGCTGGAAGTCTAATATGTATGTGATTTATTAATGCACACTAGTGTGAAACCCATGACCCATCTAATCTGTTACCTGTCCTTGGCAACGTAGTATACAGTTGTCTATGTCCAGTGATAGGATTCTACAAGGAGGATCAACAGCGTTTTAAATCTAGACATCTTCTGTTGAATTAGAACACATCAGAGAAATACTAGAGCTGTTacactctctttcttttctcttcccctcaGGCATATGTTACTACtctttctactatttttttttttttttttagcacaagtAGTTTGTCCTAGAGCAGGCTGGGTGGGTAAATGGCCTGAAATAGCCTATCATGTCATTGATTTGCTGCTCTGAGTATATTTAGTGACATAGTCATTCTGCATCACCTGTCTATGCTTGTCACTGAGTTAGAGTGGCTTGAAGCCCAGGACACACCAGTACAGAGCAGGCAAGTGACACATTCCCTgtagagagaagaaggaaaattgaaaaggaaaacagaaagctaACCATTCAGAAACTTATGCATGGAGTTAGAGCACTAACAGTGGGAGTAGAAGCTATCCACACTACCCACCCCCACTTTCCTTATCAACTCCCAAGAACATTTTTTCCCTGCAGTGTCTATGTGTCTATGCTTTGCTTTGCAAGGGTTAATTAGAGGGCCCATTATGCATCAAATTAAAGGCTTGGCTGCCTGAGAGTTCCTTTCAGAAAGGATTAGGGTCTCTGATGAGGCTTTGATGTAATAGATGGAGCTGTTC
