# Supplementary material for: LNC_003296 regulates the proliferation and differentiation of ovine myoblasts by modulating the stability of Hsp70
Source: Vet Q. 2026 Aug 25;46(1):2720646. doi: 10.1080/01652176.2026.2720646 (PMC13520862; doi:10.1080/01652176.2026.2720646)
Supplement: Supplementary file 3 [file TVEQ_A_2720646_SM6633.docx]

| Gene name | sequence (5’-3’) |
| --- | --- |
| LNC_003296 | 5’-CTCCATCTATTACATCAAAGCCTCA-3’ |
|  | 5’-GGACAGGTAACAGATTAGATGGGTC-3’ |
|  | 5’-GTGCTCCTGTTCCATGACTGC-3’ |
| 18S | 5’-CTGCCTTCCTTGGATGTGGTAGCCGTTTC-3’ |
| NC | 5’-TGCTTTGCACGGTAACGCCTGTTTT-3’ |

**Supplementary Table.1** The sequence of the FISH probe.

**Supplementary Table.2** The interference sequence of the gene.

| Gene name | Primer sequence (5’-3’) | Tm/℃ |
| --- | --- | --- |
| LNC_003296 | F: 5’- CTTACCTGCCTCCTGAGAAATC-3’  R: 5’- GACGTACTCCCTTCCCAATTT-3’ | 60 |
| *CCND1* | F: 5’-CGCAGCAGGCCACCGTTC-3’  R: 5’-GCAGCAGGAAGAGCAGATGGG-3’ | 60 |
| *CDK4* | F: 5’-TCGGCGGCTGACGGAGAC-3’  R: 5’-CAACCCAACCCTGACTCGGAATC-3’ | 60 |
| *CNOT2* | F: 5’-CACTTCACACGCCTCCATCTCC-3’  R: 5’-TTCCAATGCCCTGACCAACCTG-3’ | 60 |
| *MyHC-Ⅰ* | F: 5’-ACGCACCCTCACTTTGTACGC-3’  R: 5’-CTCTGCCGAAAGTCCCCATAG-3’ | 60 |
| *MyoD* | F: 5’-GACAGGGAGGAGGGGGTAGAG-3’  R: 5’-TGCTGTCTCAAAGGAGCAGA-3’ | 60 |
| 18S | F: 5’-TCGATGCTCTTAGCTGAGTGTCC-3’  R: 5’-GCGGTCCTATTCCATTATTCCTAGC-3’ | 60 |

| Gene name | sequence (5’-3’) |
| --- | --- |
| LNC_003296 | 5’-CTCCA+TCTAT+TACATCAAAGCCTCA-3’ |

**Supplementary** **Table.3 The sequence of the pull-down probe**
